# Acquired Thrombotic Thrombocytopenic Purpura in a Patient with Pernicious Anemia

**DOI:** 10.1155/2017/1923607

**Published:** 2017-04-04

**Authors:** Ramesh Kumar Pandey, Sumit Dahal, Kamal Fadlalla El Jack Fadlalla, Shambhu Bhagat, Bikash Bhattarai

**Affiliations:** Interfaith Medical Center, Brooklyn, NY, USA

## Abstract

*Introduction*. Acquired thrombotic thrombocytopenic purpura (TTP) has been associated with different autoimmune disorders. However, its association with pernicious anemia is rarely reported.* Case Report*. A 46-year-old male presented with blood in sputum and urine for one day. The vitals were stable. The physical examination was significant for icterus. Lab tests' results revealed leukocytosis, macrocytic anemia, severe thrombocytopenia, renal dysfunction, and unconjugated hyperbilirubinemia. He had an elevated LDH, low haptoglobin levels with many schistocytes, nucleated RBCs, and reticulocytes on peripheral smear. Low ADAMTS13 activity (<10%) with elevated ADAMTS13 antibody clinched the diagnosis of severe acquired TTP, and plasmapheresis was started. There was an initial improvement in his hematological markers, which were however not sustained on discontinuation of plasmapheresis. For his refractory TTP, he was resumed on daily plasmapheresis and Rituximab was started. Furthermore, the initial serum Vitamin B12 and reticulocyte index were low in the presence of anti-intrinsic factor antibody. So with the concomitant diagnosis of pernicious anemia, Vitamin B12 was supplemented. The rest of the immunological workups were negative. Subsequently, his symptoms resolved and his hematological parameters improved.* Discussion*. While pernicious anemia can masquerade as TTP, an actual association between the two can also occur and needs further evaluation and characterization.

## 1. Introduction

Thrombotic thrombocytopenic purpura (TTP) is a rare hematological disorder, characterized by microangiopathic hemolytic anemia (MAHA) and thrombocytopenia, leading to end organ damage like fever, renal dysfunction, and neurological manifestations [1]. The pathogenesis of TTP involves deficiency of von Willebrand factor (vWF) cleaving metalloproteinase, known as ADAMTS13 [2–4]. In congenital TTP, this deficiency is secondary to mutation in ADAMTS13 gene [5, 6]. Acquired TTP, on the other hand, results from the development of IgG autoantibodies against the enzyme and is associated with other autoimmune disorders like systemic lupus erythematosus (SLE), Hashimoto's thyroiditis, Sjögren's syndrome, and psoriasis [7–13]. However, its association with pernicious anemia has rarely been reported.

Pernicious anemia is an autoimmune disorder and classically manifests as macrocytic anemia with hypersegmented neutrophils on peripheral smear. However, multiple cases of pernicious anemia and the resulting Vitamin B12 deficiency presenting with MAHA and thrombocytopenia, and thus masquerading as TTP, have been described [14–18]. But the actual association of pernicious anemia and TTP, with the two entities being present simultaneously in a patient, has rarely been reported.

Here, we describe a case of a young man with severe acquired TTP, who was concurrently diagnosed with pernicious anemia.

## 2. Case Report

**Figure 1:**
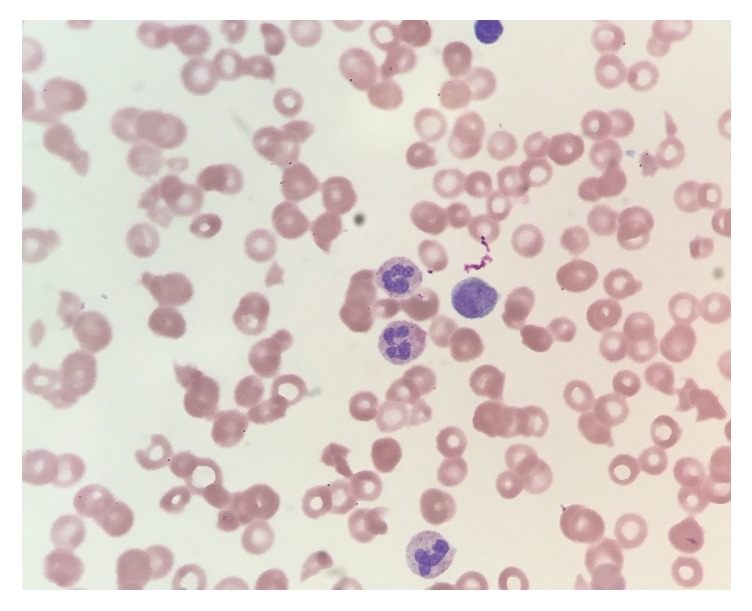
Peripheral smear.

A 46-year-old male patient with past medical history significant for mild intermittent bronchial asthma presented to our ED with blood-smeared sputum on clearing his throat and blood in urine for one day. The rest of the review of symptoms was negative. There were no similar episode in the past and no family history of any bleeding disorder or malignancy. The vitals were stable with a temperature of 98.2 F, pulse rate of 72, respiratory rate of 18, and blood pressure of 140/94 mm Hg. Physical examination was significant for icterus and absence of any petechial rash, lymphadenopathy, or hepatosplenomegaly. Lab tests' results were significant for leukocyte of 15,000/*μ*L (normal 4,500–11,000/*μ*L), hemoglobin (Hb) of 10.7 g/dL (normal 13.5–17.5 g/dL), hematocrit (Hct) of 32% (normal 41–53%), mean corpuscular volume of 102 fL (normal 80–100 fL), mean corpuscular hemoglobin of 34.2 pg (normal 26–34 pg), platelet of 13,000/*μ*L (normal 130,000–400,000/*μ*L), blood urea nitrogen of 41 mg/dL (normal 8–20 mg/dL), serum creatinine of 1.9 mg/dL (normal 0.4–1.3 mg/dL), and total bilirubin of 3.1 mg/dL (normal 0.3–1.2 mg/dL) with unconjugated bilirubin of 2.6 mg/dL (normal 0.2–1.1 mg/dL). Subsequent tests showed an elevated LDH (1499 IU/L, normal 98–192 IU/L) and low haptoglobin levels (<10 mg/dL, normal 34–200 mg/dL) with many schistocytes, nucleated RBCs, and reticulocytes (2.3%, normal 0.5–1.5%) on peripheral smear (Figure 1). ADAMTS13 activity of less than 10% (normal > 66%) with elevated ADAMTS13 antibody (>140 u/mL, normal < 12 u/mL) clinched the diagnosis of severe acquired TTP, and the patient was started on plasmapheresis.

Furthermore, in the background of macrocytic anemia and a reticulocyte index of 1.04 at presentation, which worsened to Hb of 6.9 mg/dL and Hct of 20.6% on the third day of presentation, the initial serum Vitamin B12 returned to low level (202 pg/mL, normal 211–946 pg/mL) with normal serum folate (5.9 ng/mL, normal > 3.0 ng/mL) in the presence of anti-intrinsic factor (IF) antibody. Anti-parietal cell antibodies were, however, negative. So with the concomitant diagnosis of pernicious anemia, the patient was supplemented with 1000 *μ*g of intramuscular Vitamin B12 for 7 days, beginning on the third day. The rest of the immunological workups, including antibodies against double stranded DNA, Smith antigen, thyroid peroxidase, myeloperoxidase, and proteinase-3, were negative. Thyroid function test was normal with free T4 of 1.07 ng/dL (normal 0.82–1.77 ng/dL) and thyroid stimulating hormone level of 2.38 uIU/mL (normal 0.450–4.500 uIU/mL). Daily plasmapheresis and Vitamin B12 supplementation improved the hematological markers over the subsequent days. The platelet count rose to 204,000/*μ*L and Hb and Hct stabilized at 9 mg/dL and 27%, respectively, with a reticulocyte count of 5.38% and a reticulocyte index of 2.36, while the serum LDH (302 IU/L), serum creatinine (1.4 mg/dL), and serum bilirubin (1.2 mg/dL) continued to fall. So a decision to taper the frequency of plasmapheresis was made. However, a day after skipping a session of plasmapheresis, the platelet count dropped to 67,000/*μ*L which further dropped to 22,000/*μ*L over the next few days. So a diagnosis of refractory TTP was made, and the patient was put back on daily plasmapheresis schedule. His platelet counts improved steadily to 148,000/*μ*L at which point it was decided to start the patient on Rituximab, while tapering down the frequency of plasmapheresis. He received two doses of Rituximab at a dose of 375 mg/m^2^ body surface area every week in the hospital and two more doses were planned after discharge from the hospital. Over the subsequent days, his symptoms resolved and there was significant improvement in his platelet count, hemoglobin, hematocrit, LDH, serum creatinine, and bilirubin. So after a total of 31 sessions of plasmapheresis with 24 units of fresh frozen plasma per session, the frequency of plasmapheresis was slowly tapered off, and he continued to remain asymptomatic while his hematological parameters stabilized with a platelet count of 257,000/*μ*L at discharge.

## 3. Discussion

Autoimmunity, with the development of antibodies against ADAMTS13 and the subsequent deficiency of ADAMTS13, is the driving process behind acquired TTP [7, 8]. Our patient had a very low ADAMTS13 level with elevated titers of ADAMTS13 antibodies, making it a severe form of acquired TTP. Autoimmune diseases tend to cooccur in individuals and families [19]. Cooccurrences of TTP with other autoimmune diseases have been reported. Multiple case reports have described the development of TTP in the background of SLE [9–11, 20, 21]. Similarly, there are cases of cooccurrence of Sjögren's syndrome and TTP [12, 22–24]. One particular study investigated the prevalence of concurrent autoimmune disorders in 76 patients with TTP [13]. They found a statistically significant higher occurrence of Hashimoto's thyroiditis, SLE, idiopathic thrombocytopenia purpura, psoriasis, and celiac disease in patients with TTP as compared to the general population. Our patient was simultaneously diagnosed with TTP and pernicious anemia, but this association has rarely been reported before [12].

Pernicious anemia is an autoimmune disorder that impairs the secretion and function of intrinsic factors secreted from the gastric parietal cells. The subsequent Vitamin B12 deficiency impairs DNA synthesis without affecting synthesis of cytoplasmic components [25]. The classic hematological finding in pernicious anemia consists of megaloblastic anemia, characterized by macrocytosis and hypersegmented neutrophils on peripheral smear [26–28]. However, myriad of other hematological presentations including pancytopenia and hemolytic anemia are seen [29]. Vitamin B12 deficiency results in ineffective erythropoiesis and causes intramedullary destruction of RBCs, which manifests as hemolytic anemia with indirect hyperbilirubinemia and elevated LDH. Moreover, elevated levels of homocysteine in Vitamin B12 deficiency promotes hemolysis besides causing endothelial dysfunction with microvascular thrombi formation and results in peripheral hemolysis and thrombocytopenia [30–33]. In fact, multiple cases of pernicious anemia and the resulting Vitamin B12 deficiency presenting with MAHA and thrombocytopenia with schistocytosis have been described [14–18]. These cases of isolated pernicious anemia, thus, may masquerade as TTP and pose initial therapeutic dilemma. In contrast to these previously reported cases where pernicious anemia was mistaken for TTP, our patient had concomitant pernicious anemia and TTP showing a possible association between these two autoimmune conditions. Furthermore, our patient needed a two-pronged treatment strategy as each entity had the potential to feed into the pathogenesis of another. TTP with its massive hemolysis and stimulation of erythropoiesis could worsen the Vitamin B12 deficiency of pernicious anemia, while pernicious anemia with its propensity for microvascular thrombi formation could worsen the MAHA and thrombocytopenia of TTP. Rituximab along with plasmapheresis treated the TTP in our patient, while Vitamin B12 injections treated the pernicious anemia.

## 4. Conclusion

Acquired TTP is immunologically mediated and has been associated with several autoimmune disorders like systemic lupus erythematosus (SLE) and Hashimoto's thyroiditis. While several cases of TTP-like features in patients with pernicious anemia have been reported, actual TTP in such patients is rarely reported. This association between TTP and pernicious anemia needs further evaluation given the reported overlap in features of the two entities and the potential of each entity to feed into the pathogenesis of another.

## References

[1] Moschcowitz E. (1952). An acute febrile pleiochromic anemia with hyaline thrombosis of the terminal arterioles and capillaries; an undescribed disease. *The American Journal of Medicine*.

[2] Furlan M., Robles R., Lammle B. (1996). Partial purification and characterization of a protease from human plasma cleaving von Willebrand factor to fragments produced by in vivo proteolysis. *Blood*.

[3] Hurskainen T. L., Hirohata S., Seldin M. F., Apte S. S. (1999). ADAM-TS5, ADAM-TS6, and ADAM-TS7, novel members of a new family of zinc metalloproteases. General features and genomic distribution of the ADAM-TS family. *The Journal of Biological Chemistry*.

[4] Tsai H. M. (1996). Physiologic cleavage of von Willebrand factor by a plasma protease is dependent on its conformation and requires calcium ion. *Blood*.

[5] Furlan M., Robles R., Solenthaler M., Wassmer M., Sandoz P., Lammle B. (1997). Deficient activity of von Willebrand factor-cleaving protease in chronic relapsing thrombotic thrombocytopenic purpura. *Blood*.

[6] Levy G. G., Nichols W. C., Lian E. C. (2001). Mutations in a member of the ADAMTS gene family cause thrombotic thrombocytopenic purpura. *Nature*.

[7] Tsai H. M., Lian E. C. (1998). Antibodies to von Willebrand factor-cleaving protease in acute thrombotic thrombocytopenic purpura. *The New England Journal of Medicine*.

[8] Furlan M., Robles R., Galbusera M. (1998). von Willebrand factor-cleaving protease in thrombotic thrombocytopenic purpura and the hemolytic-uremic syndrome. *The New England Journal of Medicine*.

[9] Beigelman P. M. (1951). Variants of the platelet thrombosis syndrome and their relationship to disseminated lupus. *AMA Archives of Pathology*.

[10] Dekker A., O'Brien M. E., Cammarata R. J. (1974). The association of thrombotic thrombocytopenic purpura with systemic lupus erythematosus: a report of two cases with successful treatment of one. *The American Journal of the Medical Sciences*.

[11] Kosloske A. M., Pisciotta A. V. (1961). The syndrome of thrombotic thrombocytopenic purpura as the presenting manifestation in systemic lupus erythematosus. *Marquette Medical Review*.

[12] Steinberg A. D., Green W. T., Talal N. (1971). Thrombotic thrombocytopenic purpura complicating Sjogren's syndrome. *The Journal of the American Medical Association*.

[13] John M.-L., Scharrer I. (2012). Autoimmune disorders in patients with idiopathic thrombotic thrombocytopenic purpura. *Hamostaseologie*.

[14] Walter K., Vaughn J., Martin D. (2015). Therapeutic dilemma in the management of a patient with the clinical picture of TTP and severe B12 deficiency. *BMC Hematology*.

[15] Tadakamalla A. K., Talluri S. K., Besur S. (2011). Pseudo-thrombotic thrombocytopenic purpura: a rare presentation of pernicious anemia. *North American Journal of Medical Sciences*.

[16] Abbott D. W., Friedman K. D., Karafin M. S. (2016). Differentiation of pernicious anemia from thrombotic thrombocytopenic purpura: the clinical value of subtle pathologic findings. *Transfusion and Apheresis Science*.

[17] Malla M., Seetharam M. (2016). To treat or not to treat: a rare case of pseudo-thrombotic thrombocytopenic purpura in a Jehovah's Witness. *Transfusion*.

[18] Panchabhai T., Patil P., Riley E., Mitchell C. (2016). When the picture is fragmented: vitamin B12 deficiency masquerading as thrombotic thrombocytopenic purpura. *International Journal of Critical Illness and Injury Science*.

[19] Heward J., Gough S. C. L. (1997). Genetic susceptibility to the development of autoimmune disease. *Clinical Science*.

[20] Changcharoen B., Bolger D. T. (2015). Thrombotic thrombocytopenic purpura as an initial presentation of systemic lupus erythematosus with acquired ADAMTS 13 antibody. *BMJ Case Reports*.

[21] Abu-Hishmeh M., Sattar A., Zarlasht F. (2016). Systemic Lupus erythematosus presenting as refractory thrombotic thrombocytopenic purpura: a diagnostic and management challenge. a case report and concise review of the literature. *American Journal of Case Reports*.

[22] Toumeh A., Josh N., Narwal R., Assaly R. (2014). Refractory thrombotic thrombocytopenic purpura associated with primary sjogren syndrome treated with rituximab: a case report. *American Journal of Therapeutics*.

[23] Abe H., Tsuboi N., Yukawa S. (2004). Thrombotic thrombocytopenic purpura complicating Sjögren's syndrome with crescentic glomerulonephritis and membranous nephritis. *Modern Rheumatology*.

[24] Schattner A., Friedman J., Klepfish A. (2002). Thrombotic thrombocytopenic purpura as an initial presentation of primary Sjögren's syndrome. *Clinical Rheumatology*.

[25] Yoshida Y., Todo A., Shirakawa S., Wakisaka G., Uchino H. (1968). Proliferation of megaloblasts in pernicious anemia as observed from nucleic acid metabolism. *Blood*.

[26] Koury M. J., Price J. O., Hicks G. G. (2000). Apoptosis in megaloblastic anemia occurs during DNA synthesis by a p53-independent, nucleoside-reversible mechanism. *Blood*.

[27] Healton E. B., Savage D. G., Brust J. C. M., Garrett T. J., Lindenbaum J. (1991). Neurologic aspects of cobalamin deficiency. *Medicine*.

[28] Thompson W. G., Cassino C., Bahitz L. (1989). Hypersegmented neutrophils and vitamin B_12_ deficiency. Hypersegmentation in B_12_ deficiency. *Acta Haematologica*.

[29] Andrès E., Affenberger S., Zimmer J. (2006). Current hematological findings in cobalamin deficiency. A study of 201 consecutive patients with documented cobalamin deficiency. *Clinical and Laboratory Haematology*.

[30] Olinescu R., Kummerow F. A., Handler B., Fleischer L. (1996). The hemolytic activity of homocysteine is increased by the activated polymorphonuclear leukocytes. *Biochemical and Biophysical Research Communications*.

[31] Ventura P., Panini R., Tremosini S., Salvioli G. (2004). A role for homocysteine increase in haemolysis of megaloblastic anaemias due to vitamin B12 and folate deficiency: results from an in vitro experience. *Biochimica et Biophysica Acta*.

[32] Zittan E., Preis M., Asmir I. (2007). High frequency of vitamin B12 deficiency in asymptomatic individuals homozygous to MTHFR C677T mutation is associated with endothelial dysfunction and homocysteinemia. *American Journal of Physiology—Heart and Circulatory Physiology*.

[33] Nappo F., De Rosa N., Marfella R. (1999). Impairment of endothelial functions by acute hyperhomocysteinemia and reversal by antioxidant vitamins. *Journal of the American Medical Association*.

